# Genomic insights into *Dactylosporangium* sp. AC04546, a rubber degrader with three latex clearing proteins

**DOI:** 10.3389/fmicb.2024.1378082

**Published:** 2024-05-30

**Authors:** Ann Anni Basik, Namiko Gibu, Yukimura Kawagiwa, Siuk-Mun Ng, Tiong Chia Yeo, Kumar Sudesh, Daisuke Kasai

**Affiliations:** ^1^Sarawak Biodiversity Centre, Kuching, Sarawak, Malaysia; ^2^Department of Materials Science and Bioengineering, Nagaoka University of Technology, Nagaoka, Japan; ^3^Codon Genomics S/B, Seri Kembangan, Selangor, Malaysia; ^4^Ecobiomaterial Research Laboratory, School of Biological Sciences, Universiti Sains Malaysia, Gelugor, Penang, Malaysia

**Keywords:** rubber, degradation, actinobacteria, genome, *Dactylosporangium*

## Abstract

With more than 100 rubber-degrading strains being reported, only 9 Lcp proteins isolated from *Nocardia, Gordonia, Streptomyces, Rhodococcus, Actinoplanes*, and *Solimonas* have been purified and biochemically characterized. A new strain, Dactylosporangium sp. AC04546 (strain JCM34239), isolated from soil samples collected in Sarawak Forest, was able to grow and utilize natural or synthetic rubber as the sole carbon source. Complete genome of Strain AC04546 was obtained from the hybrid assembly of PacBio Sequel II and Illumina MiSeq. Strain AC04546 has a large circular genome of 13.08 Mb with a G+C content of 72.1%. The genome contains 11,865 protein-coding sequences with 3 latex clearing protein (*lcp*) genes located on its chromosome. The genetic organization of the *lcp* gene cluster is similar to two other reported rubber-degrading strains—*Actinoplanes* sp. OR16 and Streptomyces sp. CFMR 7. All 3 Lcp from strain AC04546 were expressed in *Escherichia coli* and exhibited degrading activity against natural rubber. The distinctiveness of strain AC04546, along with other characterized rubber-degrading strains, is reported here.

## 1 Introduction

Rubber manufacturing and production remain strong year after year due to high market demand; the amount of natural rubber produced in 2015 doubled compared to 2000 (Leong et al., 2023). Rubber products are resistant to natural degradation due to the vulcanization process and the presence of additives, antioxidants, and fillers, which take thousands of years to degrade naturally. Over the years, rubber products (e.g., tires) have been taking up landfills, releasing toxic chemicals into the environment, becoming breeding grounds for mosquitoes, and increasing the risk of fire hazards. Recent findings reveal that products that either consist of or contain high levels of rubber had the worst toxicity impact on the microorganisms investigated (Sørensen et al., 2023).

Green technology, such as biodegradation, is important to ensure the success of “responsible consumption and production” under Sustainable Development Goal 12. A safer alternative is needed to discard, process, or convert rubber wastes into high–value-added products. Rubber biodegradation refers to the degradation and devulcanization of rubber products by microorganisms growing on the rubber surface. These microorganisms exert their effect by adhering to the rubber surface or secreting enzymes that degrade the surroundings. Unlike conventional methods of burning or landfilling, rubber biodegradation does not produce harmful and toxic side products. Complex rubber molecules are converted into small oxidized derivatives used as carbon and energy sources by microorganisms (Soares and Steinbüchel, 2022). Both Gram-positive and Gram-negative bacteria have rubber-degrading enzymes, either latex-clearing protein (Lcp) or rubber oxygenase A (RoxA) and B (RoxB). Although structurally different, the enzymes are dioxygenases that catalyze oxidative cleavage of the *cis* double bonds in the poly (*cis*-1,4-isoprene) backbone, generating carbonyl and aldehyde end groups (Birke et al., 2017). RoxA and RoxB are key enzymes in rubber degradation by Gram-negative bacteria.

Well-studied rubber-degrading bacteria mostly belong to the Gram-positive group Actinobacteria, known to secrete Lcp. The group includes strains of genera such as *Streptomyces, Nocardia, Gordonia, Rhodococcus*, and *Actinoplanes*. We previously reported a newly identified rubber-degrading strain, *Dactylosporangium* sp. AC04546, which was identified from a group of 940 actinobacteria (Basik et al., 2021). Those strains were screened for their ability to produce clearing zones on latex overlay agar, which is an indication of rubber-degrading enzymes being secreted (Basik et al., 2021).

*Dactylosporangium* is a genus first discovered in 1967 (Thiemann et al., 1967), and only 17 species have been reported to date, classifying it as a rare genus that is less frequently isolated. Strain AC04546 was isolated from a soil sample collected in a secondary forest near a large Aglaia tree in Sarawak, Malaysia. The tree and its sap may have influenced the evolution of this rubber-degrading strain. Here, we report the complete genome of strain AC04546 compared to the genomes of other related rubber degraders. To date, eight incomplete and five complete *Dactylosporangium* genomes have been submitted to the GenBank database. The complete genome size ranges from 8.221 to 11.96 Mb. Strain AC04546 has the largest genome at 13.08 Mb, and an *in-silico* comparison classifies it as a distinct species. We are interested in this strain for its ability to utilize natural and vulcanized rubber as the sole carbon source. Strain AC04546 grew well in the presence of tire samples as the sole carbon and energy source, showing visible changes on the tire surface seen in scanning electron microscope (SEM) images and changes in the Attenuated Total Reflection-Fourier Transform Infrared (ATR-FTIR spectra) within 30 days of incubation (Basik et al., 2021).

Apart from strain AC04546, strains with 3 *lcp* genes on their chromosomes have been reported for *Streptomyces* sp. CFMR 7 (Nanthini et al., 2017) and *Actinoplanes* sp. OR16 (NBRC 114529). *Actinoplanes* sp. OR16 was isolated from a botanical garden in Japan (Imai et al., 2011), and the *lcp* genes have been functionally characterized (Gibu et al., 2020). Here, we aim to characterize strain AC04546 with reference to other rubber-degrading strains in which Lcp enzymes have been characterized.

## 2 Materials and methods

### 2.1 Growth condition of *Dactylosporangium* sp. AC04546

*Dactylosporangium* sp. AC04546 is a mesophilic strain that grows well at 28°C and up to 45°C on yeast malt extract (ISP2) agar. This strain is a slow grower; when cultivated in an ISP2 broth at 28°C, 180 rpm, its exponential growth rate is from day 8 to 13 and reaches a stationary phase on day 14.

### 2.2 Whole genome sequencing

Cultivation and genomic extraction were conducted using methods previously described (Basik et al., 2021). Genomic DNA was sheared to a size distribution of ~20 kb and used directly for SMRTbell library construction using the SMRTbell^®^ Express Template Preparation Kit 2.0 (Pacific Biosciences, Menlo Park, CA, USA). Subsequently, digestion of ssDNA ends, DNA damage repair, end repair, and ligation of adapters were performed. The SMRTbell libraries were purified using AMPure^®^PB and size-selected to remove hairpin and adapter dimers. DNA fragments without adapters were removed using exonucleases. The size selected SMRTbell libraries were loaded onto the PacBio Sequel II platform according to the manufacturer's instructions (Pacific Biosciences, Menlo Park, CA, USA). Sequencing was performed using the Sequel II Sequencing Kit 2.0 (Pacific Biosciences, Menlo Park, CA, USA).

Subreads generated from the PacBio Sequel II were assembled using Flye v2.9 with default parameters and “–pacbio-raw” for PacBio reads. Quast v3.1 (Gurevich et al., 2013) was used to assess the contiguity of the genome assembly, while BUSCO v4.1.4 (Simão et al., 2015), actinobacteria_class_odb10 profile was used to assess the completeness of the genome. Additionally, short-read data generated from Illumina MiSeq were recruited to further improve the assembly. The short-read sequences were trimmed using Trimmomatic version 0.38 (Bolger et al., 2014) to remove adapters and low-quality (Phred <20) and short reads (<50 bp). Gapfiller v1.10 was used to identify and close the gap in the original assembly with parameters: -m 29 -o 15 -r 0.8 -n 20 -t 10 -T. The gap-filled assembly was polished with the clean short-read data using Pilon v1.24 with default parameters. Circlator v1.5.5 was used to circularize the final assembly with parameters: -b2r_length_cutoff 10,000. The circularized assembly was assessed again using Quast and BUSCO, as mentioned above. A circular plot was plotted using ClicO FS, an online server available at http://103.47.253.210:3000/.

### 2.3 Phylogenetic analyses

The culture and genome of strain AC04546 were analyzed through methods described in Basik et al. (2021). The results were analyzed by the TYGS (Meier-Kolthoff et al., 2022) for (i) determination of closely related type strains, (ii) pairwise comparison of genome sequences, and (iii) phylogenetic inference.

### 2.4 Genome structural annotation

The complete genome was structurally annotated wherein protein-coding genes or CDS, tRNAs, and rRNAs were predicted from the genome using Prodigal v2.6.3 with parameters: -c -m; tRNAscan-SE v1.3.1 with default parameters; and rnammer v1.2 (Lagesen et al., 2007) with parameters: -S bac and -multi respectively. After gene prediction, the full repertoire of peptide sequences (≥33 aa) was assessed for its completeness using BUSCO v4.1.4 against the Actinobacteria_class_odb10 profile. The predicted peptide sequences annotated in the genome assembly were searched against RefSeq and SwissProt protein database using DIAMOND binary v2.0.13 (Buchfink et al., 2015) and BLAST+ v2.10.1 (Camacho et al., 2009) with parameters: -max_target_seqs 20 -max_hsps 1 -evalue 1e-5, respectively. The peptide sequences were further functionally annotated through orthology assignment using eggnog-mapper v2.0.0 based on eggNOG 5.0 (Huerta-Cepas et al., 2017, 2019). The peptides were also functionally characterized with protein domains and families using InterProScan v5.44-79.0 (Jones et al., 2014).

### 2.5 Comparative genome analyses

The newly assembled genome of strain AC04546 (GCF_022095675) and genome sequences of nine additional strains of interest (GCF_024268275, GCF_014647515, GCF_001278095, GCF_020826795, GCF_004001265, GCF_000523235, GCF_000247715, GCF_003104955, and GCF_020885655) were downloaded from NCBI RefSeq database and included in the analysis. All ten genome sequences were assessed for their contiguity and completeness using Quast v3.1 and BUSCO v4.1.4 with parameters as previously described for strain AC04546. Next, genes present or absent from selected genomes of interest were identified using Roary v3.7.0 with parameters: -i 70 -e -n -v -r -p 4. Operons were predicted for all ten genomes using Operon-Mapper, an online web server available at https://biocomputo.ibt.unam.mx/operon_mapper/.

### 2.6 Data mining

BLASTP v2.10.0+ was used to search for homologs of the reference genes of interest with a general e-value cut-off of 1e-05. Next, a manual assessment of the Blast results was carried out. Integrative Genomics Viewer (IGV) v2.10.2 was used to visualize the rubber-degrading and adjacent genes. Protein-coding genes (CDS) and open reading frame (ORF) on the genome were analyzed and viewed using Rapid Annotation of Microbial Genomes using Subsystems Technology (RAST) (https://rast.nmpdr.org/) and SnapGene Viewer (https://www.snapgene.com/). Molecular weights were also determined using SnapGene Viewer.

Pairwise sequence alignment (PSA) with Matrix EBLOSUM62 using EMBOSS Needle (Madeira et al., 2022) was made for strain AC04546 lcp amino acid sequences (https://www.ebi.ac.uk/jdispatcher/psa/emboss_needle). Lcp 3D structure and identity match were predicted using NCBI BLAST+ Protein similarity search using AlphaFold DB (https://alphafold.ebi.ac.uk/). Multiple sequence alignment of amino acids was viewed using Jalview 2.11 (Martin et al., 2020).

### 2.7 Expression of *lcp* genes in *E*. *coli*

The primers were designed using In-Fusion Cloning Primer Design Tool v1.0 (https://www.takarabio.com/learning-centers/cloning/primer-design-and-other-tools) with pCold IV as the destination vector and NdeI as the restriction digest. The *lcp* sequence without twin-arginine translocation (TAT) signal sequence were added as inserts. TAT sequence regions were identified using SignalP 5.0 software (https://services.healthtech.dtu.dk/services/SignalP-5.0/) (Almagro et al., 2019).

The coding regions of *lcp1, lcp2*, and *lcp3* were independently amplified by PCR using lcp1_F and R, lcp2_F and R, and lcp3_F and R primer pairs:

**Table:** 

**Oligo nucleotide**	**Sequence 5′-3′**
Lcp1_F	TCGAAGGTAGGCATATG-GCGCCCGCCGCCGCGGGCACC
Lcp1_R	GTACCGAGCTCCATATG-TCAGGCCCGGTTCCCGGTGG
Lcp2_F	TCGAAGGTAGGCATATG-CCGAGCGGCTCGGTCGCCG
Lcp2_R	GTACCGAGCTCCATATG-TCAGGACGGGCGGTGGCGACC
Lcp3_F	TCGAAGGTAGGCATATG-TGGTCGCCGAGCGGCTCG
Lcp3_R	GTACCGAGCTCCATATG-TCACTCGGGGCGGTTCATGG

The forward primer was added with an NdeI site to the start codon of the target gene. Amplified fragments were purified using the Fast Gene Plasmid Mini Kit. They were separately cloned into a pCold IV vector, which was subsequently digested with NdeI by using an In-fusion HD Cloning Kit (Takara Bio Inc., Tokyo, Japan).

The resultant plasmids were independently introduced into *E. coli* Rosetta-gami B(DE3)pLysS and the transformants were grown at 37°C in Luria Bertani (LB) medium containing ampicillin (100 mg/mL), kanamycin (25 mg/mL), chloramphenicol (25 mg/mL), and tetracycline (10 mg/mL). When the absorbance at 600 nm (A600) of the culture reached 0.5, 1 mM isopropyl-β-D-thiogalactopyranoside (IPTG) was added, and the cultures were further incubated at 15°C for 24 h. The resulting cells were harvested and resuspended in 50 mM phosphate buffer (pH 7.4). The cells were disrupted using an ultrasonic disrupter, and the lysates were centrifuged. The supernatant was collected, treated with streptomycin sulfate 10%, and filtered through a 0.45 μm filter.

The supernatant was then purified using the ÄKTA^TM^ Start Protein Purification System with a HiTrap TALON Crude 1 mL Superflow column (Cytiva, Uppsala, Sweden) previously equilibrated with 50 mM sodium phosphate (pH 7.4) containing 300 mM NaCl. Proteins were allowed to bind for 1 min at 4°C under rotation, followed by washing five times in 5 mL of the same buffer. His-tagged proteins were eluted with 5 mL of 50 mM sodium phosphate (pH 7.4) containing 300 mM NaCl and 150 mM imidazole. The fractions were pooled and concentrated using 30 kDa Amicon (R) Ultra Centrifugal filters.

### 2.8 Enzyme assay

To test whether the Lcp expressed by recombinant *E*.*coli* pCold IV::*lcp* strains can degrade poly (*cis*-1,4-isoprene) into aldehydes, crude extracts of Lcp1, 2, and 3 were applied onto a 0.4% deproteinized natural rubber (DPNR)-overlay agar plate and incubated overnight at 30°C. Three mL of Schiff's reagent was applied onto the plate and left for 30 min at room temperature. Destaining was carried out using 5 mL of 10% sodium hydrogen sulfide.

## 3 Results and discussion

### 3.1 Whole genome sequencing and assembly

The whole genome of *Dactylosporangium* sp. AC04546 was sequenced using the PacBio Sequel II platform. The total throughput for the sample was ~115.2 k raw reads or ~1.277 Gb of data, with 98 × genome coverage. A circularized genome comprising one single contig with a total genome size of 13.08 Mb and an average GC content of 72.1% was generated from the hybrid assembly (Genbank submission CP139171). No plasmid was detected. The circularized genome was estimated to achieve 98.3% completeness based on the Actinobacteria class odb10 profile. A total of 11,865 coding sequences (>99 bp), 9 rRNAs, and 83 tRNAs were annotated to the genome (Table 1). The coding sequences (CDS) were categorized into 14% (1,616 CDS) with subsystem categories and 86% (10,740 CDS) without subsystem categories. A subsystem is a set of functional roles that together implement a specific biological process or structural complex (Overbeek et al., 2014). Strain AC04546 has subsystems such as the metabolism of aromatic compounds and sulfur metabolism, which is an added advantage as a rubber degrader should the enzymes be expressed.

**Table 1:** Genome assembly and annotation statistics of strain AC04546.

	**Circularized genome of strain AC04546**
Number of contigs	1
Total assembly size (bp)	13,083,175
GC%	72.10
Circular	Yes
Genome completeness (profile: actinobacteria_class_odb10)	C:98.3%[S:97.3%,D:1.0%],F:0.3%,M:1.4%,n:292
Number of genes	11,957
CDS (>99 bp)	11,865
rRNA	9
tRNA	83
Proteome completeness (profile: actinobacteria_class_odb10)	C:98.3%[S:97.3%,D:1.0%],F:0.3%,M:1.4%,n:292

C, complete BUSCOs; S, complete and single-copy BUSCOs; D, complete and duplicated BUSCOs; F, fragmented BUSCOs; M, missing BUSCOs; n, number of BUSCO groups searched.

The coding sequences were functionally annotated to four databases, namely the RefSeq, SwissProt, EggNOG v5.0.0, and InterPro v79.0, with 98.55% of proteins having at least one functional annotation (Table 2).

**Table 2:** Functional annotation statistics of strain AC04546.

**Database**	**Details**	**Circularized genome of strain AC04546**	
	Total number of proteins	11,865	100.00%
RefSeq	Number of proteins with RefSeq blast hit	11,557	97.39%
SwissProt	Number of proteins with SwissProt blast hit	7,595	64.00%
EggNOG	Number of proteins with EggNOG ortholog matched	10,906	91.90%
InterPro	Number of proteins with InterPro entry matched	10,428	87.87%
	Number of proteins with ≥1 annotation	11,693	98.55%

### 3.2 Phylogenetic analyses

**Figure 1:**
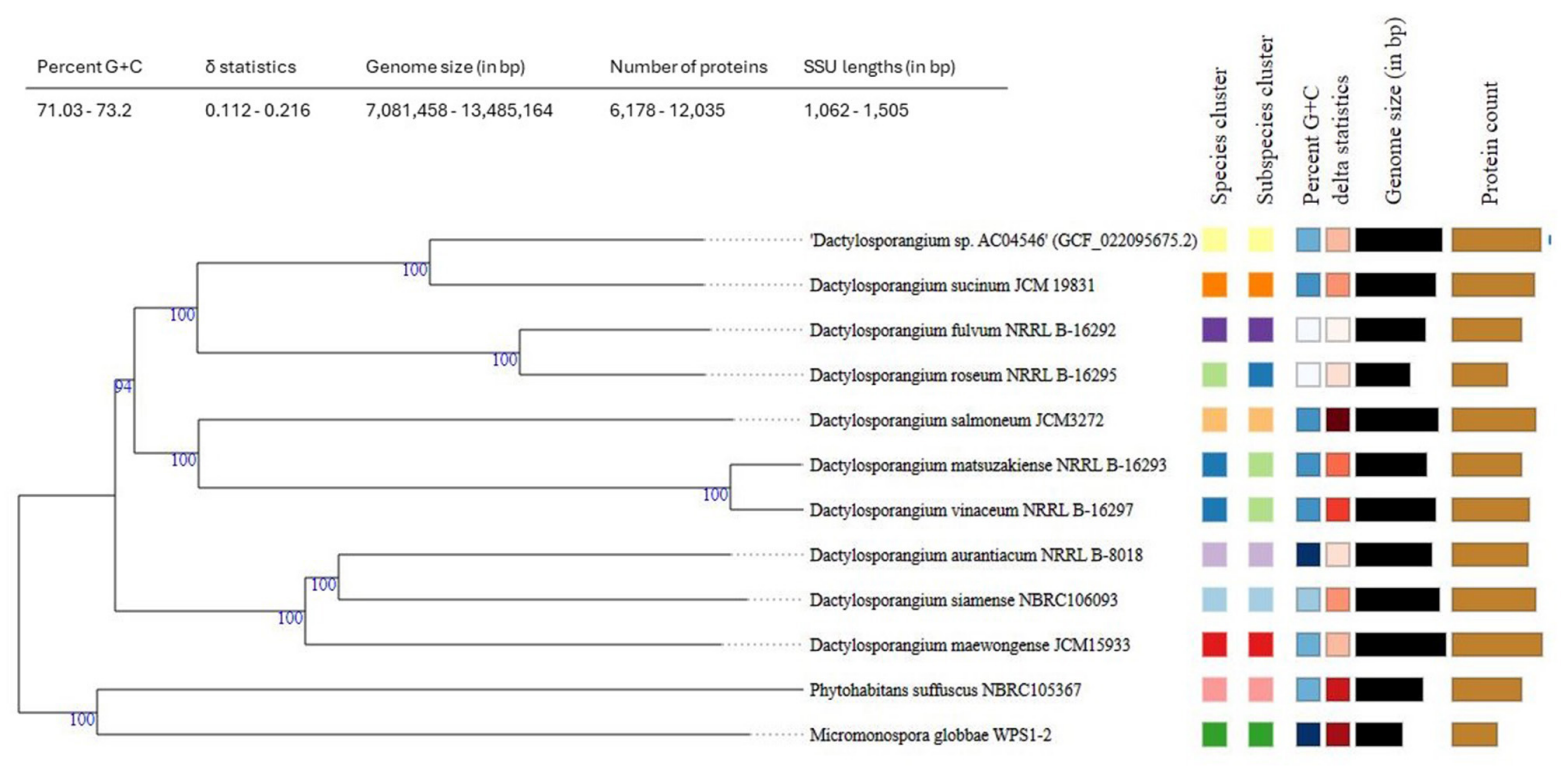
Tree inferred with FastME 2.1.6.1 (Lefort et al., 2015) from Genome BLAST Distance Phylogeny (GBDP) distances calculated from strain AC04546 and closely related strains genome sequences. The branch lengths are scaled in terms of the GBDP distance formula d5. The numbers above the branches are GBDP pseudo-bootstrap support values > 60% from 100 replications, with an average branch support of 98.6%. The tree was rooted at the midpoint (Farris, 1972).

Based on its complete genome, strain AC04546 is closely matched to *D. sucinum* JCM19831, a strain isolated from a soil sample collected from a peat swamp forest soil in Rayong Province, Thailand (Phongsopitanun et al., 2015). There are no reports of *D. sucinum* for its rubber-degrading activity or the presence of *lcp* genes. Genome-based phylogeny shows that they are within the same clade (TYGS, https://tygs.dsmz.de) (Figure 1). Bacterial species are defined as similar species when they present more than 95% average nucleotide identity (ANI) and 70% of digital DDH (dDDH) compared to another species in the same genus (Thompson et al., 2013; Volpiano et al., 2021). Digital ddH for strain AC04546 was at only 52.4%, and an ANI value of 91.51% was obtained. Therefore, strain AC04546 is a separate species from *Dactylosporangium sucinum*.

### 3.3 Comparative genome analyses

In addition to the current genome sequence, a total of nine additional strains of interest comprising the reference strain *D. sucinum*, seven strains belonging to type 1 with latex-clearing protein genes (*Streptomyces* sp. AC04842, *Streptomyces* sp. CFMR 7, *Microtetraspora* sp. AC03309, *Actinoplanes* sp. OR16, *Gordonia polyisoprenivorans* VH2, *Nocardia nova* SH22a, and *Rhodococcus* sp. RDE2), and one strain belonging to type 2 with *roxA* and *roxB* genes (*Steroidobacter cummioxidans* strain:35Y), were included for comparative analysis. The genome sequences downloaded from the NCBI RefSeq database consist of assemblies at the complete, scaffold, and contig levels, with genome sizes ranging from 5.72 to 13.08 Mb and contig or scaffold numbers ranging from 1 to 579. Based on the BUSCO Actinobacteria class odb10 profile, the genome completeness ranged from 75.7 to 100.0% (Table 3). These rubber-degrading strains were isolated from samples collected in Japan, Vietnam, and Brazil. Isolated samples ranged from soil, plant roots, and wastes from rubber processing facilities.

**Table 3:** Sequence metrics and features of ten genomes of interest.

	**Subject**	**Reference**	**Type 1**							**Type 2**
			**(with** ***lcp*** **genes)**							**(with *roxA* and *roxB***
										**genes)**
**Family**	**Micromonosporaceae**			**Streptomycetaceae**		**Streptosporangiaceae**	**Nocardiaceae**		**Gordoniaceae**	**Steroidobacteraceae**
Strain	*Dactylosporangium* sp. AC04546	*Dactylosporangium sucinum*	*Actinoplanes* sp. OR16	*Streptomyces* sp. AC04842	*Streptomyces* sp. CFMR 7	*Microtetraspora* sp. AC03309	*Nocardia nova* SH22a	*Rhodococcus* sp. RDE2	*Gordonia polyisoprenivorans* VH2	*Steroidobacter cummioxidans* 35Y
Country of origin	Sarawak, Malaysia	Thailand	Japan	Sarawak, Malaysia	Penang, Malaysia	Sarawak, Malaysia	Brazil	Vietnam	Vietnam	Japan
Isolation source	Soil, secondary forest	Soil, peat swamp forest	Soil, botanical garden	Soil, national park	Rubber plantation	Soil, secondary forest	Root*, Couma macrocarpa*	Waste, rubber-processing factory	Soil, rubber tree plantation	Unknown
Accession	GCF_022095675	GCF_014647515	GCF_004001265	GCF_024268275	GCF_001278095	GCF_020826795	GCF_000523235	GCF_020885655	GCF_000247715	GCF_003104955
Rubber-degrading enzyme expressed and characterized	Expressed	No	Yes	No	No	No	Yes	Yes	Yes	Yes
Assembly level	Complete	Scaffold	Complete	Contig	Complete	Contig	Complete	Complete	Complete	Contig
# contigs	1	221	1	579	2	448	1	7	2	126
# contigs (≥1,000 bp)	1	195	1	427	2	230	1	7	2	126
# contigs (≥10,000 bp)	1	138	1	233	2	138	1	4	2	112
# contigs (≥50,000 bp)	1	77	1	30	2	69	1	3	2	57
Largest contig	13,083,175	346,783	9,293,892	105,598	8,207,742	226,531	8,348,532	5,386,255	5,669,805	336,437
Total length	13,083,175	12,103,263	9,293,892	7,764,148	8,307,279	9,335,430	8,348,532	5,715,406	5,844,299	7,936,208
Total length (≥1,000 bp)	13,083,175	12,083,779	9,293,892	7,663,967	8,307,279	9,187,175	8,348,532	5,715,406	5,844,299	7,936,208
**Family**	**Micromonosporaceae**			**Streptomycetaceae**		**Streptosporangiaceae**	**Nocardiaceae**		**Gordoniaceae**	**Steroidobacteraceae**
Total length (≥10,000 bp)	13,083,175	11,890,307	9,293,892	6,818,573	8,307,279	8,902,433	8,348,532	5,699,004	5,844,299	7,836,986
Total length (≥50,000 bp)	13,083,175	10,181,094	9,293,892	2,064,878	8,307,279	7,027,899	8,348,532	5,699,004	5,844,299	6,383,457
N50	13,083,175	134,443	9,293,892	33,342	8,207,742	88,541	8,348,532	5,386,255	5,669,805	97,490
N75	13,083,175	74,216	9,293,892	16,471	8,207,742	50,534	8,348,532	5,386,255	5,669,805	55,538
L50	1	29	1	74	1	36	1	1	1	22
L75	1	60	1	156	1	69	1	1	1	49
GC (%)	72.10	72.36	70.34	71.89	72.03	70.04	67.77	67.86	66.98	61.72
#Ns	-	9,484	-	-	-	-	-	-	-	194
#N per 100 kbp	-	78	-	-	-	-	-	-	-	2
BUSCO actinobacteria_ class_odb10	C:98.3% [S:97.3%, D: 1.0%], F: 0.3%, M: 1.4%, n: 292	C:97.6% [S:96.6%, D:1.0%], F:0.3%, M:2.1%, n:292	C:98.6% [S:96.9%, D:1.7%], F:0.3%, M:1.1%, n:292	C:99.3% [S:99.0%, D:0.3%], F:0.7%, M:0.0%, n:292	C:100.0% [S:98.3%, D:1.7%], F:0.0%, M:0.0%, n: 292	C:99.7% [S:97.3%, D:2.4%], F:0.3%, M:0.0%, n:292	C:100.0% [S:99.0%, D:1.0%], F:0.0%, M:0.0%, n: 292	C:100.0% [S:100.0%, D:0.0%], F:0.0%, M:0.0%, n: 292	C:99.3%[S:99.3%, D:0.0%], F:0.0%, M:0.7%, n:292	C:75.7% [S:73.3%, D:2.4%], F:5.1%, M:19.2%, n: 292

C, complete BUSCOs; S, complete and single-copy BUSCOs; D, complete and duplicated BUSCOs; F, fragmented BUSCOs; M, missing BUSCOs; n, number of BUSCO groups searched.

As most strains are taxonomically distant, the gene presence/absence analysis resulted in a total of 62,369 orthologous gene clusters for the ten strains based on a 70% identity cut-off, with 80.47% of them unique to only one strain. Through operon prediction, a total of 2,996–6,605 operons were predicted for the ten strains, which appear to correlate with their genome sizes.

**Figure 2:**
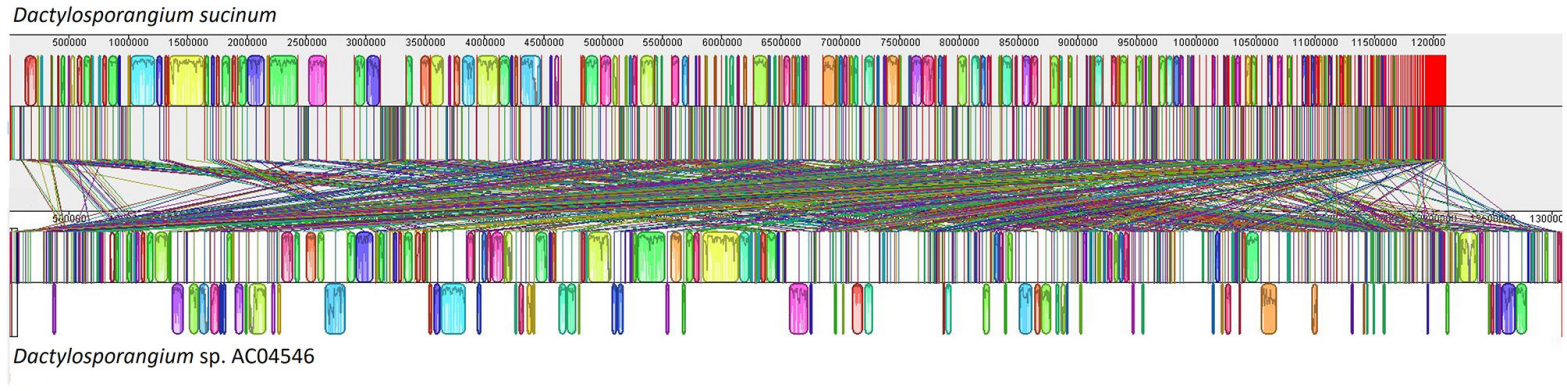
Genome comparison between strain AC04546 and *Dactylosporangium sucinum*. The alignment display for each genome sequence is organized in a horizontal panel together with the scale showing the sequence coordinates of that genome. Each block outlines the regions of the genome sequence aligned to a portion of another genome. The block above the center line indicates that the aligned region is in the forward orientation relative to the first genome sequence. In contrast, the block below the center line shows that the aligned region is in the inverse orientation. The same-colored blocks indicate the segments, or Locally Collinear Blocks (LCBs), that are conserved among the two bacteria compared.

Based on Figure 2, a comparative genome analysis between strain AC04546 and the closely related genome of the *Dactylosporangium sucinum* genome showed that the genome organization is very different. This is clearly depicted by the same-colored blocks, which indicate the segments, or Locally Collinear Blocks (LCBs), that are conserved among the bacteria compared (linked by the same line). On the other hand, areas that are completely white were not aligned and possibly contained sequence elements specific to a particular genome.

### 3.4 Gene mining and visualization

**Figure 3:**
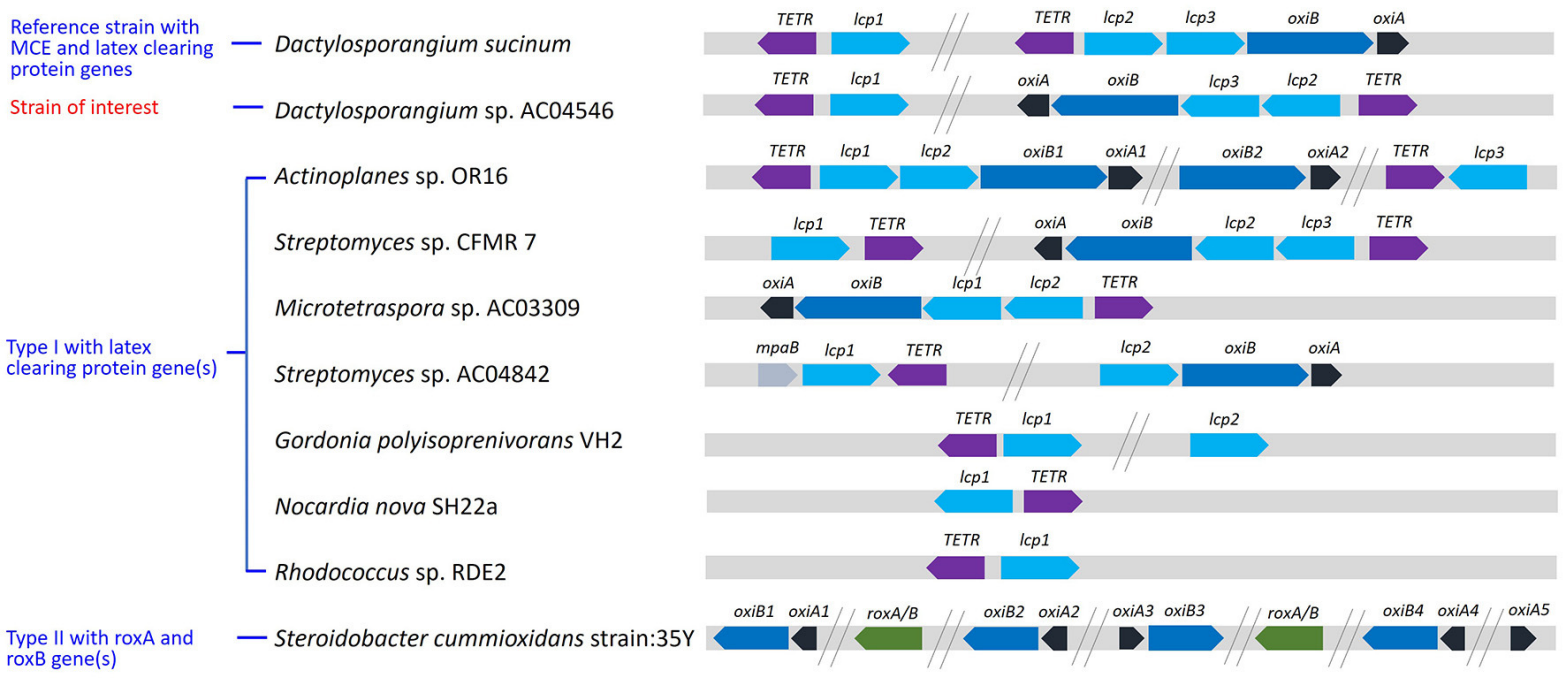
Schematic representation of operon involved in rubber degradation across ten selected strains.

Operons involved in rubber degradation across ten strains were compared by mining the latex clearing protein (*lcp*) genes, its adjacent genes isoquinoline 1-oxidoreductase subunit alpha (*oxiA*), isoquinoline 1-oxidoreductase subunit beta (*oxiB*) and the rubber oxygenase genes (*roxA* and *roxB*) (Figure 3). The number of *lcp* genes detected for all ten strains of interest is consistent with the previous reports. Strain AC04546 (Basik et al., 2021), *Streptomyces* sp. CFMR 7 (Nanthini et al., 2017), and *Actinoplanes* sp. OR16 (Gibu et al., 2020) contained three copies of *lcp* genes, while *Streptomyces* sp. AC04842 (Basik et al., 2022), *Microtetraspora* sp. AC03309 (Basik et al., 2022), and *Gordonia polyisoprenivorans* VH2 (Hiessl et al., 2012) were found to contain two copies of *lcp*. *Nocardia nova* SH22a (Luo et al., 2014) and *Rhodococcus* sp. RDE2 (Gibu et al., 2022) contained only one *lcp* gene each. On the other hand, the Gram-negative *Steroidobacter cummioxidans* strain 35Y (Sharma et al., 2018) was found to only contain *roxA* and *roxB* but not *lcp*.

The *oxiA* and *oxiB* genes were not detected for strains containing mycolic acid, including *Nocardia, Gordonia*, and *Rhodococcus*. Mycolic acid-containing strains produce biofilms where rubber-degrading enzymes are being secreted into; biofilms enable the microbial cells to attach onto hydrophobic surfaces such as rubber (Linos et al., 2000). Clear zone formers, on the other hand, have mycelia which penetrates the rubber surfaces and secrete rubber-degrading enzymes through the mycelial corridors (Röther et al., 2017). There could be a correlation between OxiAB and the presence or absence of clear zones. It has been reported that in *Nocardia* and *Gordonia*, which do not generate clear zones, aldehyde dehydrogenase (ALDH) is involved instead of OxiAB. Whereby OxiAB is thought to oxidize oligomers extracellularly because it contains TAT signal sequences, but the ALDH have differences that function intracellularly (Rose et al., 2005; Suzuki et al., 2022). It was suggested that the OxiAB enzyme is required for the clear-zone formation on natural rubber in strain AC04546.

**Figure 4:**
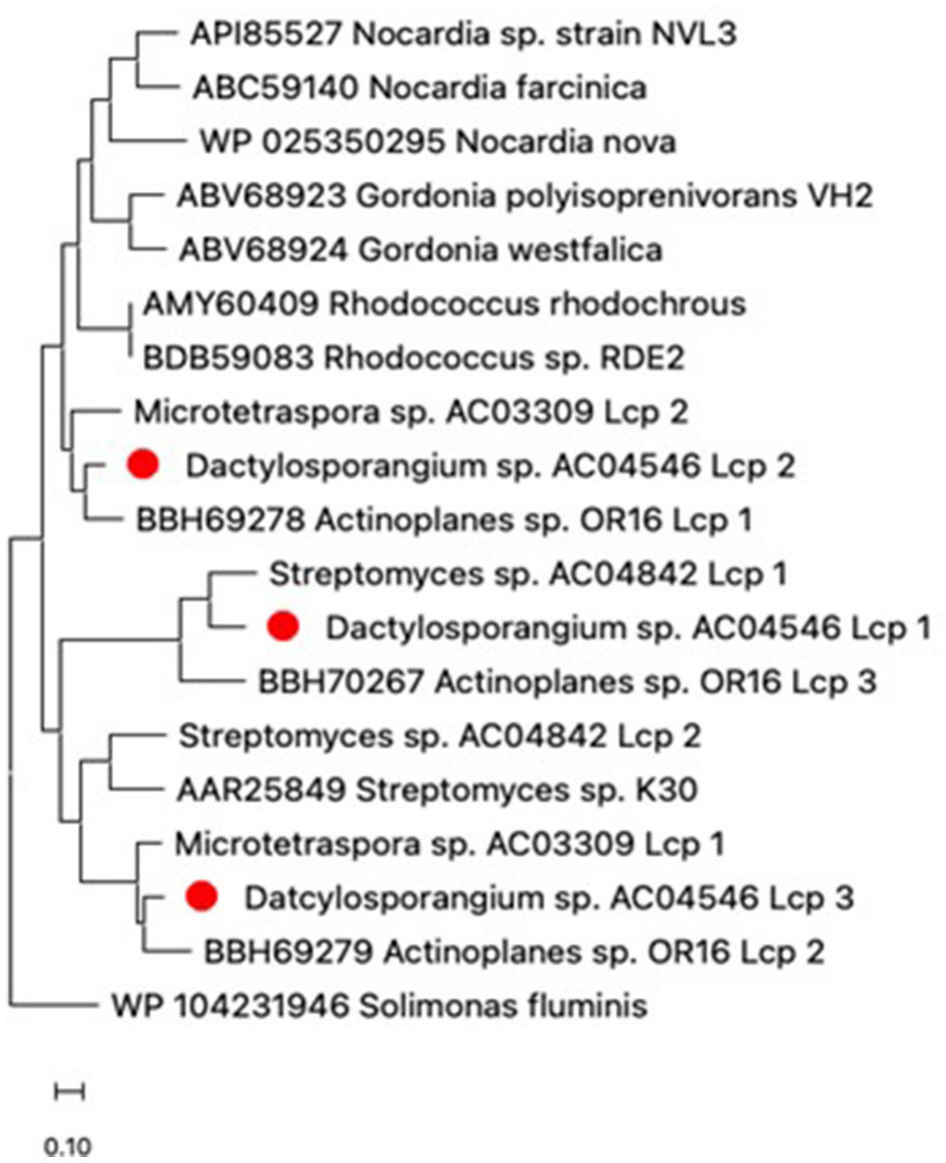
Phylogenetic distribution of amino acid sequences of Lcp from strain AC04546 (red dot), *Streptomyces* sp. AC04842, *Microtetraspora* sp. AC03309 and characterized Lcps from the NCBI database. The Lcp amino acid sequence from *Solimonas fluminis* (Gram-negative strain) was used as an outgroup. The tree was constructed using maximum likelihood, MEGA11 (Tamura et al., 2021). The bar below the phylogenetic trees represents the scale of sequence divergence.

Evolutionary differences of Lcps between strain AC04546 and other Actinomycetes such as *Streptomyces* sp. AC04842 and *Microtetraspora* sp. AC03309 was characterized. The amino acid sequences of lcp1, lcp2, and lcp3 for strain AC04546 were closely related to lcp3, lcp1, and lcp2 of *Actinoplanes* sp. OR16, respectively (Figure 4). This trend was also observed in the estimates of evolutionary divergence between the lcp sequences (Supplementary Table 1).

**Figure 5:**
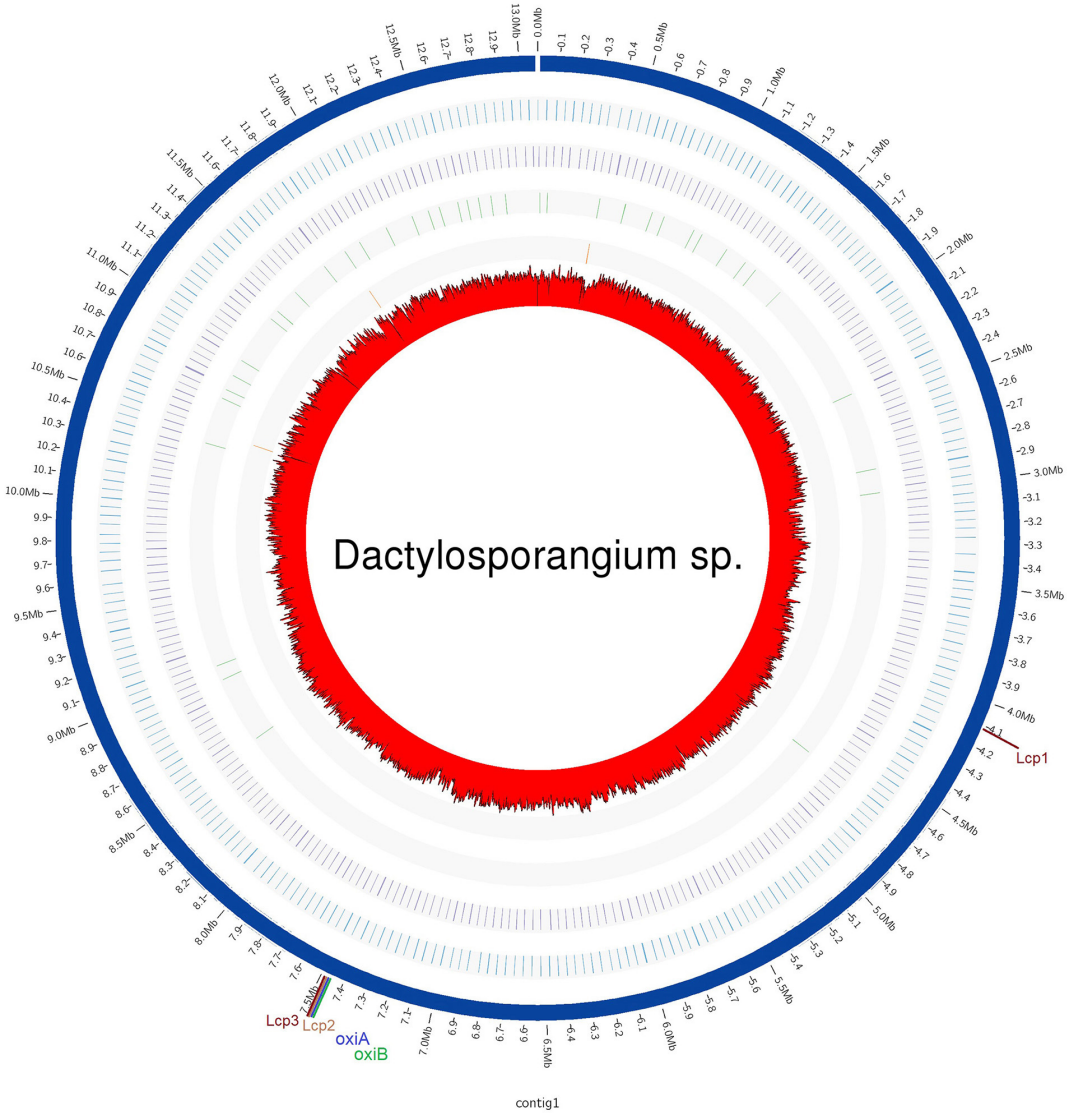
Circular representation of strain AC04546 genome and features. The genome sequence is shown at the outermost ring. The G+C content is indicated in the center of the figure. The second and third outermost rings show the rRNAs and tRNAs, respectively. Locations of latex-clearing protein (*lcp*) genes on the genome are shown.

For strain AC04546, the location and size of nucleotide and amino acid sequences of the related genes are summarized in Table 4. Based on the gene's location, the *lcp3, lcp2*, and *oxiB-oxiA* genes are considered part of an operon. The amino acid sequence identity of Lcp1-Lcp2, Lcp2-Lcp3, and Lcp1-Lcp3 is 49.2%, 45.9%, and 60.8%, respectively (Supplementary Table 2). The gene cluster, including *lcp3, lcp2*, and *oxiB*-*oxiA* of strain AC04546, is similar to previously reported strains containing the 3 *lcp* genes: *Streptomyces* sp. CFMR 7 (Nanthini et al., 2017) and *Actinoplanes* sp. strain OR16 (Gibu et al., 2020), where two *lcp* genes are located adjacent to each other, followed by *oxiB-oxiA* genes, with the other *lcp* gene located far apart (Figure 4). The complete genome and *lcp* genes for strain AC04546 were visualized and are illustrated in Figure 5. The *lcp1* gene is located about 3.4 Mb apart from the *lcp2, lcp3*, and *oxiB-oxiA* gene*, s*imilar to the draft genome reported in Basik et al. (2021). No specific MCE-family genes were detected in the complete genome.

**Table 4:** The location and size of nucleotide and amino acid sequences of the related genes in *Dactylosporangium* sp. AC04546.

**Gene**	**Accession number**	**Length (bp)**	**Amino acids (aa)**	**Theoretical mass (kDa)**	**TAT signal peptide cleavage site**	**RBS position (from the start codon)**
*lcp1*	MW659700	1,221	406	44.2	40–41 aa (ALA-AP)	6 bp upstream
*lcp2*	MW659701	1,209	402	43.8	30–31 aa (AWT-WA)	9 bp upstream
*lcp3*	MW659702	1,224	407	44.9	30–31 aa (AWS-WS)	9 bp upstream
*oxiA*	MZ726745	477	158	16.8	-	-
*oxiB*	MZ751063	2,283	760	80.3	-	-

The amino acid sequence of oxiA and oxiB strain AC04546 showed 80.5% (100% query coverage, QC) and 79.3% (97% QC) identities with oxiA (ACTI_59660) and oxiB (ACTI_59650) of *Actinoplanes* sp. OR16, respectively. The amino acid sequence of oxiA and oxiB strain AC04546 showed 74.5% (100% QC) and 77.5% (98% QC) identities with oxiA (AAR26467) and oxiB (AAR26466) of *Streptomyces* sp. K30 respectively (Rose et al., 2005). Based on the similarity of these sequences, oxiA and oxiB are thought to be involved in the oxidation of oligo isoprene aldehyde in strain AC04546.

**Figure 6:**
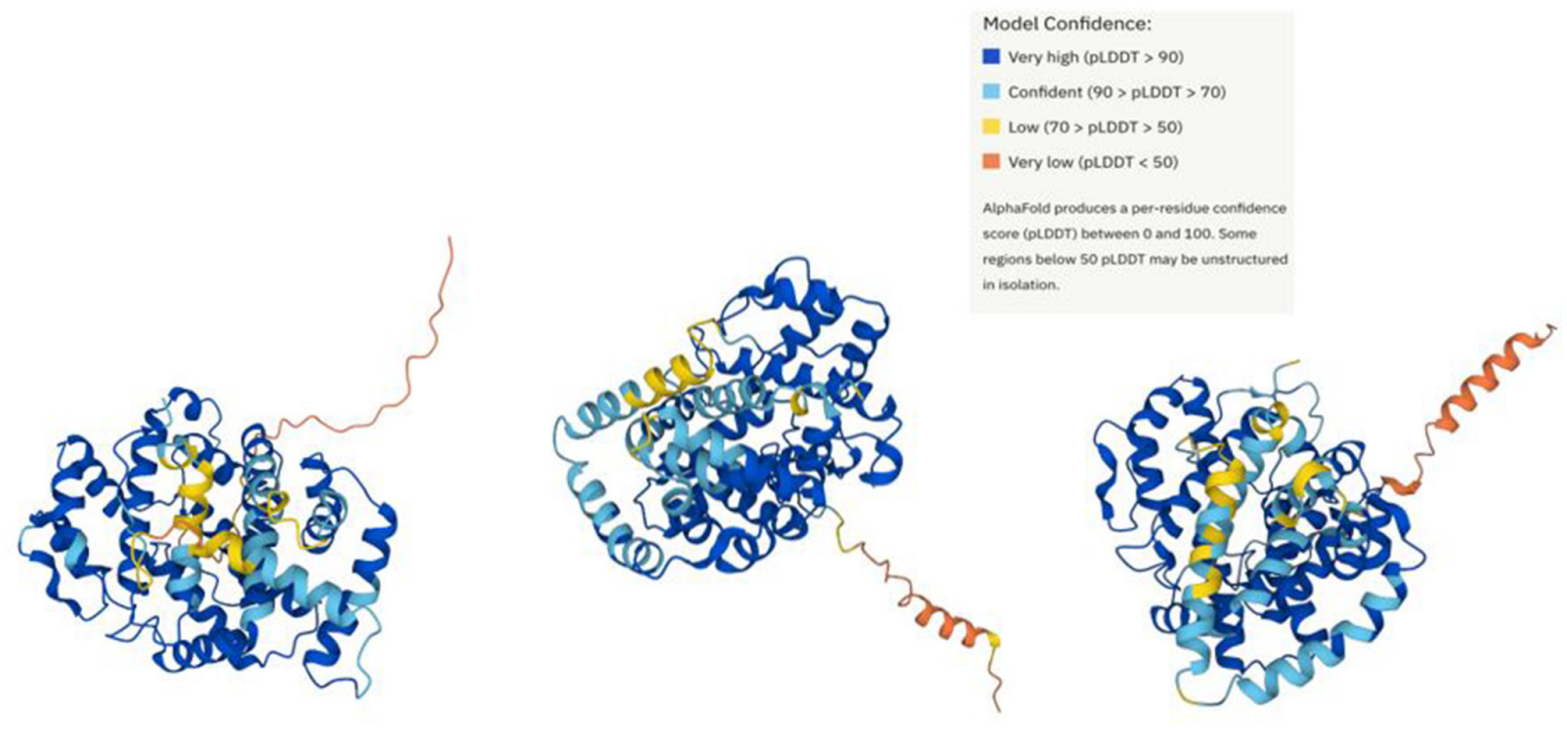
Prediction of strain AC04546 Lcp1, Lcp2, and Lcp3 structure (left to right) using AlphaFold Database.

Lcp1, Lcp2, and Lcp3 structures were predicted using the AlphaFold Database (Figure 6) (Varadi et al., 2024). The structural and functional analysis of Lcp from *Streptomyces* sp. K30 identified important residues contributing to the stability of the protein, especially Arg195 and Arg202, while conserved residues Arg164, Tr168, and His198, located close to the haem cofactor, are crucial active site residues (Ilcu et al., 2017). Multiple sequence alignment of amino acid sequences of Lcps from AC04546, OR16, and K30 confirms that Lcps from AC04546 and OR16 have the conserved residues Arg164, Tr168, and His198 and 13-residue-long highly conserved region (Supplementary Figure 1) (Röther et al., 2016). It was suggested that these residues are required for the activity of these Lcps.

A 1,209 bp gene (located from 4,125,595 bp to 4,126,803 bp) that appears to encode a TetR-type transcriptional regulator is located upstream of the *lcp1* gene. It has been reported that TetR-type transcriptional regulators are involved in the regulation of Lcp expression in strain OR16 (Gibu et al., 2020), *Streptomyces coelicolor* A3(2) (Coenen et al., 2019) and *G. polyisoprenivorans* VH2 (Oetermann et al., 2019). The genes encoding TetR-type transcriptional regulators were found to be located in the vicinity of the *lcp* gene. These facts suggest that TetR-type regulators expressed from genes located in the vicinity of *lcp1* might be involved in the regulation of the *lcp* expression in strain AC04546.

### 3.5 Heterologous expression of Lcp in *E. coli*

The coding regions of each *lcp* gene were amplified by PCR and introduced into pCold IV to fuse a 6x histidine tag at the N terminal of the genes. The *lcp* genes of strain AC04546 were not well expressed in *E. coli* BL21(DE3); therefore, pCold IV:*lcp* was transformed into *E. coli* Rosetta-gami B(DE3)pLysS. Haem-containing proteins, including Lcp have been reported to be brown to red in color (Watcharakul et al., 2016). The dark yellow coloration of the cells of the transformant and supernatant expression of each *lcp* gene product was observed, but crude Lcp-his could not be purified in this system (Supplementary Figures 2–4). The histidine tag at the N-terminal may cause ineffective binding during Immobilized-metal affinity chromatography (IMAC) purification.

### 3.6 Enzyme assay

**Figure 7:**
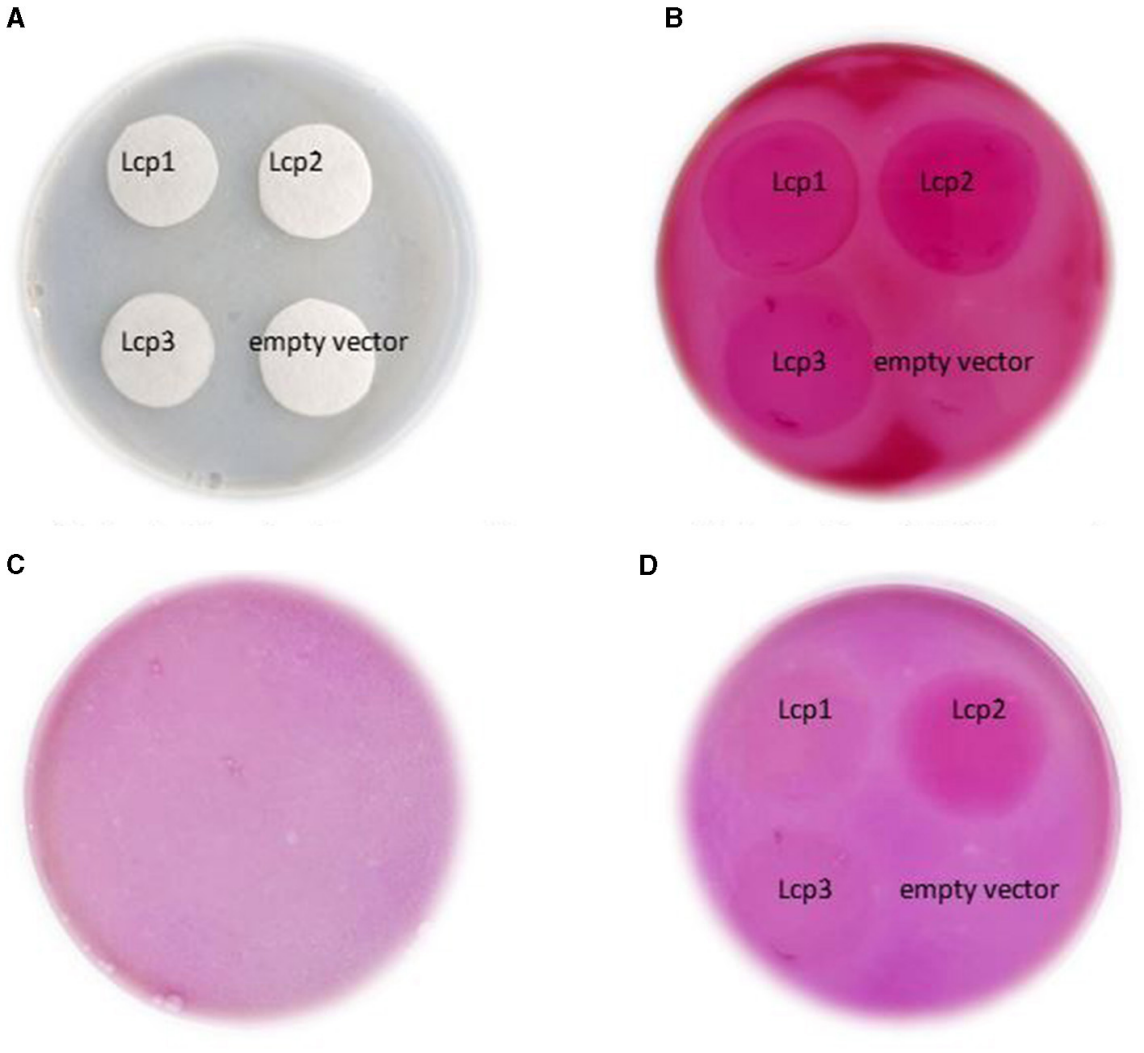
Detection of strain AC04546 Lcp1, Lcp2, and Lcp3 enzyme activity obtained from heterologous expression of *E. coli* for using Schiff's reagent for the presence of aldehyde; control plate (left) and test samples plate (right). **(A)** Application of crude enzyme on filter paper. **(B)** Application of Schiff's reagent. **(C)** Control plate (without any samples). **(D)** After destaining process.

To confirm the enzymatic activity of recombinant Lcp, crude Lcp-his extract of 5 mg/mL was spotted on DPNR-overlay agar and stained with Schiff's reagent. The appearance of purple regions at Lcp1, Lcp2, and Lcp3 positions indicates that aldehyde is produced by the reaction of Lcp from poly(*cis*-1,4-isoprene). Lcp2 showed more intense purple coloration compared to Lcp1 and Lcp3 (Figure 7). The coloration was not found when the crude extract of *E. coli* was used with an empty vector. These results strongly suggested that these Lcp can degrade poly(*cis*-1,4-isoprene) into an oligomer containing an aldehyde group by adding the oxygen molecule.

Pairwise sequence alignment of the strain AC04546 and *Actinoplanes* sp. OR16 lcp amino acid sequences were conducted. The sequences of Lcp1, Lcp2, and Lcp3 from AC04546 showed 62.6%, 80.1%, and 76.4% identities with those of Lcp3, Lcp1, and Lcp2 from *Actinoplanes* sp. OR16, respectively. It has been reported that Lcp1 has the highest activity to degrade poly(*cis*-1,4-isoprene) in *Actinoplanes* sp. OR16 (Gibu et al., 2020). Based on the sequence similarity, it was suggested that these Lcp enzymes from strain AC04546 have the activity to degrade poly(*cis*-1,4-isoprene), and Lcp2 has the highest activity in strain AC04546 (see Section 3.4).

## 4 Conclusion

*Dactylosporangium* sp. AC04546, a newly discovered species, has three Lcp enzymes that are expressed. Lcp proteins of this strain did not match other known Lcp in the database, and AlphaFold prediction of all three structures of Lcp1, Lcp2, and Lcp3 differ from each other. Differences in degradation capability were also observed during the enzyme assay. Moreover, the expression levels of the three Lcp were different, with Lcp2 being more highly expressed. This is the first report of strain AC04546 and its Lcp enzymes compared to rubber-degrading enzymes from other known species. The complete genome of this strain is a useful reference for future characterization works.

## Data availability statement

The datasets presented in this study can be found in online repositories. The names of the repository/repositories and accession number(s) can be found below: NCBI - PRJNA896744.

## Author contributions

AB: Writing—original draft. NG: Investigation, Supervision, Writing—review & editing. YK: Investigation, Writing—review & editing, Supervision. S-MN: Formal analysis, Investigation, Methodology, Visualization, Writing—review & editing. TY: Funding acquisition, Project administration, Writing—review & editing. KS: Conceptualization, Project administration, Supervision, Writing—review & editing. DK: Funding acquisition, Methodology, Project administration, Supervision, Writing—review & editing.
