# Supplementary material for: Genomic insights into Dactylosporangium sp. AC04546, a rubber degrader with three latex clearing proteins
Source: Front Microbiol. 2024 May 30;15:1378082. doi: 10.3389/fmicb.2024.1378082 (PMC11169899; doi:10.3389/fmicb.2024.1378082)
Supplement: Supplementary file 1 [file Presentation_1.pdf]

Supplementary Figures & Tables

Supplementary Table 1. Estimates of evolutionary divergence between lcp sequences in this study and Lcp sequences that their Lcps has been characterized.

		1	2	3	4	5	6	7	8	9	10	11	12	13	14	15	16	17	18	19
1	*Streptomyes* AC04842_lcp_1																			
2	*Streptomyes* AC04842_lcp_2	76%																		
3	*Microtetraspora* AC03309_lcp_1	78%	49%																	
4	*Microtetraspora* AC03309_lcp_2	84%	56%	53%																
5	*Dactylosporangium* AC04546_lcp_1	29%	77%	79%	80%															
6	*Dactylosporangium* AC04546_lcp_2	69%	50%	45%	26%	68%														
7	*Dactylosporangium* AC04546_lcp_3	76%	51%	17%	55%	75%	50%													
8	AAR25849_*Streptomyces*_sp._K30	75%	37%	54%	57%	72%	51%	50%												
9	BBH69278_*Actinoplanes*_sp._OR16	70%	53%	51%	30%	64%	19%	54%	57%											
10	BBH69279_*Actinoplanes*_sp._OR16	75%	53%	26%	65%	76%	59%	22%	56%	61%										
11	BBH70267_*Actinoplanes*_sp._OR16	46%	77%	73%	81%	42%	67%	73%	74%	68%	73%									
12	ABV68923_*Gordonia_polyisoprenivorans*_VH2	83%	64%	64%	43%	84%	39%	64%	64%	42%	66%	82%								
13	ABV68924_*Gordonia_westfalica*	86%	59%	65%	46%	87%	42%	65%	63%	45%	68%	85%	25%							
14	WP025350295_*Nocardia_nova*	84%	65%	69%	51%	86%	46%	67%	64%	50%	71%	85%	44%	44%						
15	AMY60409_*Rhodococcus_rhodochrous*	82%	56%	57%	36%	76%	33%	55%	56%	31%	61%	78%	38%	42%	46%					
16	API85527_*Nocardia*_sp._strain_NVL3	81%	62%	64%	40%	80%	36%	63%	60%	39%	71%	87%	44%	41%	41%	41%				
17	ABC59140_*Nocardia_farcinica*	80%	60%	59%	40%	76%	33%	58%	61%	36%	67%	79%	42%	45%	44%	41%	29%			
18	BDB59083_*Rhodococcus*_sp._RDE2	82%	56%	57%	36%	76%	33%	55%	56%	31%	61%	78%	38%	42%	46%	0%	41%	41%		
19	WP104231946_*Solimonas_fluminis*	85%	72%	67%	59%	77%	49%	67%	64%	53%	69%	81%	56%	58%	63%	54%	65%	60%	54%	

The number of amino acid substitutions per site from between sequences is shown. Analyses were conducted using the Poisson correction model. This analysis involved 19 amino acid sequences. All ambiguous positions were removed for each sequence pair (pairwise deletion option). There was a total of 485 positions in the final dataset. Evolutionary analyses were conducted in MEGA11 (Tamura et al. 2021).

Supplementary Table 2. Pairwise sequence alignment (PSA) of strain AC04546 and Actinoplanes sp. strain OR16 latex clearing protein (lcp) amino acid sequences.

	**Strain AC04546 lcp1**				**Strain AC04546 lcp2**				**Strain AC04546 lcp3**			
	**Length (bp)**	**Gaps (%)**	**Identity (%)**	**Similarity (%)**	**Length (bp)**	**Gaps (%)**	**Identity (%)**	**Similarity (%)**	**Length (bp)**	**Gaps (%)**	**Identity (%)**	**Similarity (%)**
**Strain OR16 lcp1**	422	9.2	50.0	64.7	407	3.2	80.1	87.7	408	4.4	56.6	73.0
**Strain OR16 lcp2**	417	9.4	44.1	58.5	408	6.1	52.9	68.9	403	4.5	76.4	84.1
**Strain OR16 lcp3**	422	8.1	62.6	75.1	412	4.4	50.2	66.3	411	4.6	47.2	62.0

Note: Highlighted cells show the highest similarity among the 3 lcp amino acid sequences.

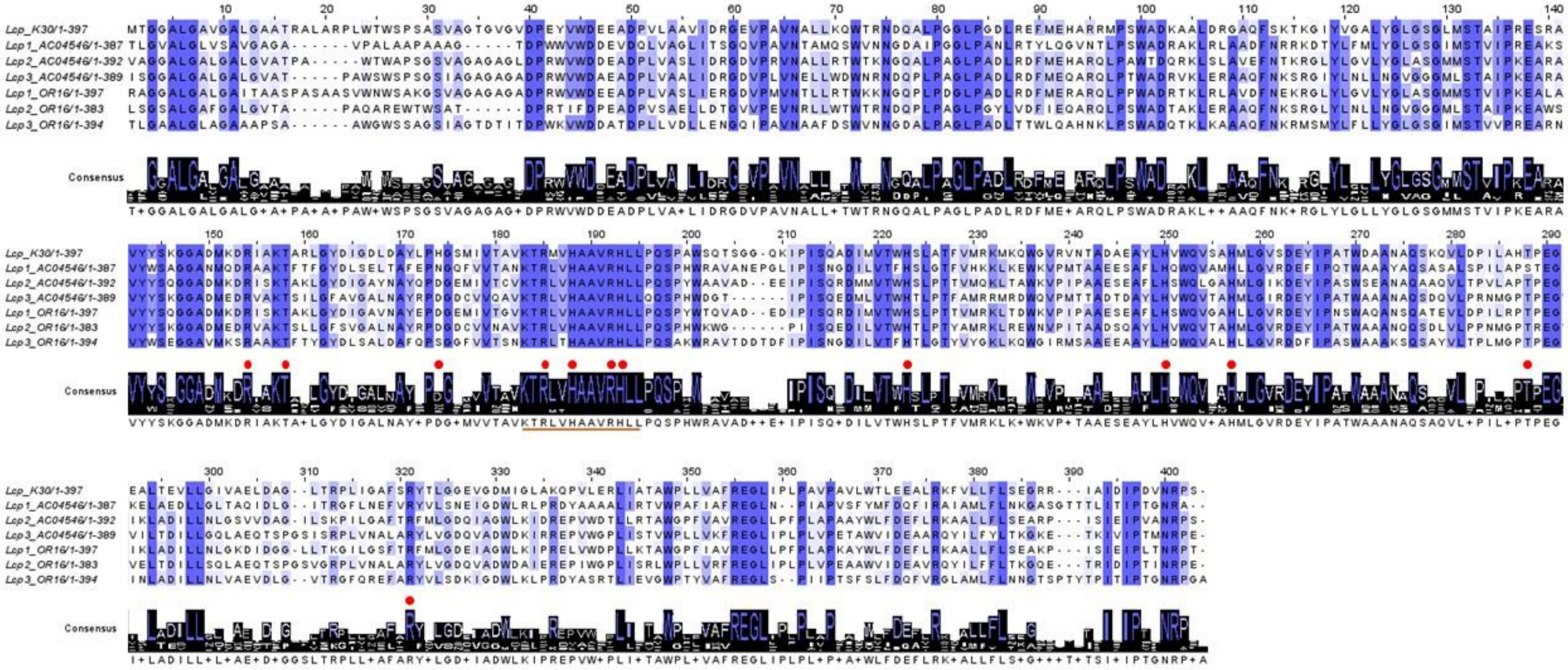

Supplementary Figure 1. Multisequence alignment of amino acid sequences for *Dactylosporangium* sp. AC04546, *Actinoplanes* sp. OR16 and *Streptomyces* sp. K30. The values of conservation (as percentages) for the residues chosen in this study (red dot) are given below. A 13-residue-long highly conserved region is indicated by a red bar. The degree of conservation of a specific residue position is indicated by the height of the respective column.

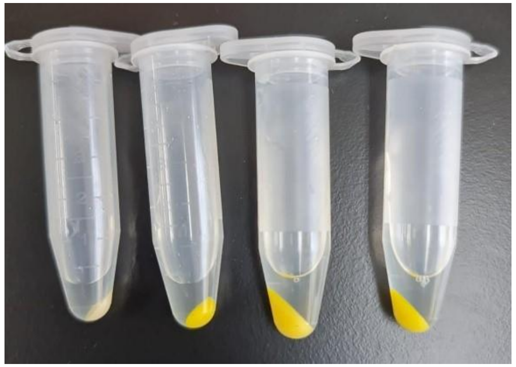

Supplementary Figure 2. The cells of *E. coli* Rosetta-gami B(DE3)pLysS with (i) empty vector and (ii) Lcp1 (iii) Lcp2 and (iv) Lcp3 expression vectors (left to right).

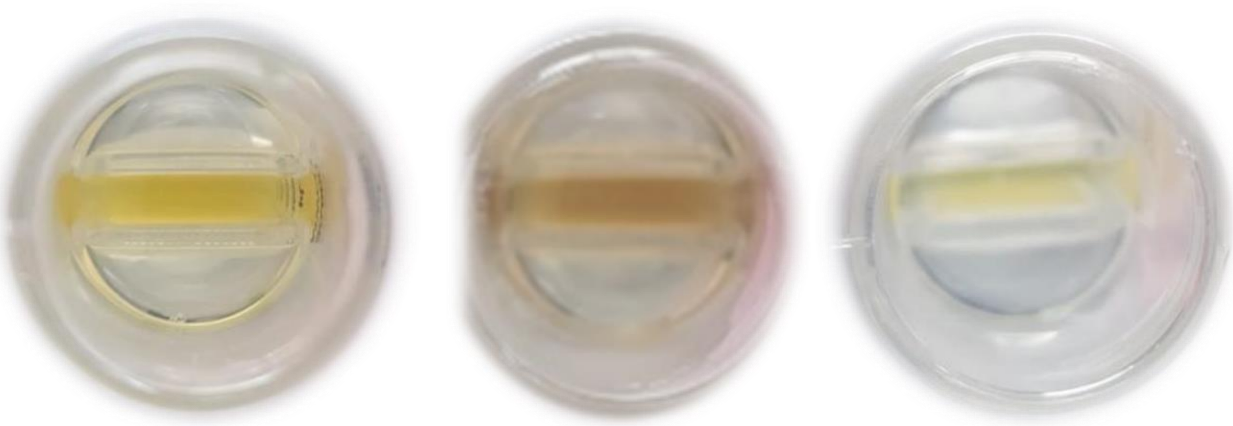

Supplementary Figure 3. Concentrated supernatant using 30 kDa filtration (left to right): Lcp1 dark yellow (14 mg/mL), Lcp2 brownish (17 mg/mL) and Lcp3 light yellow (8 mg/ mL).

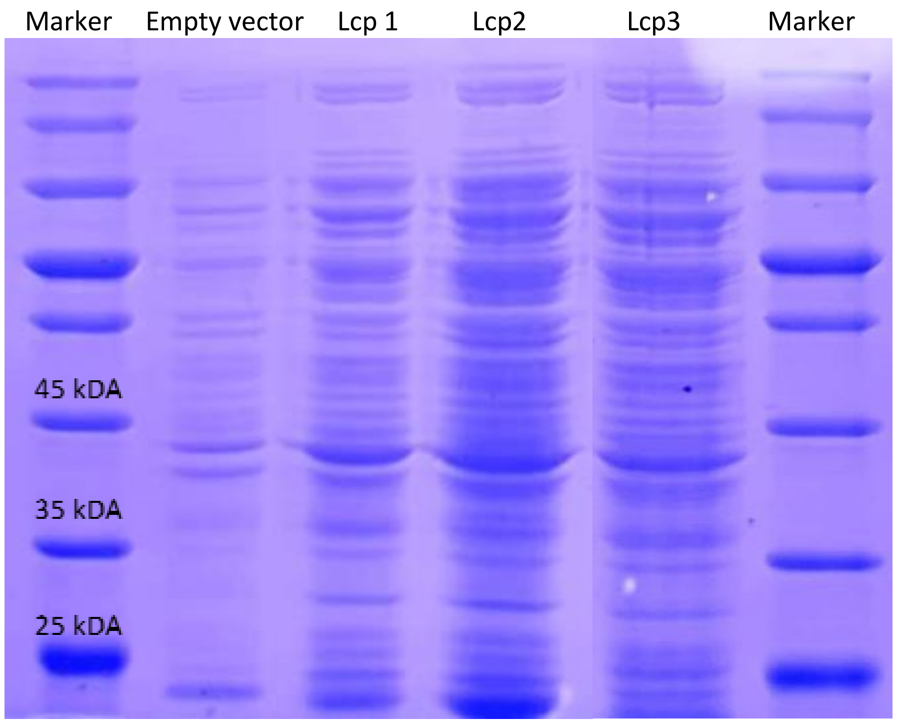

Supplementary Figure 4. SDS-PAGE of 10 mg crude Lcp-his extract, Lcp is predicted at approximately 45 kDa.

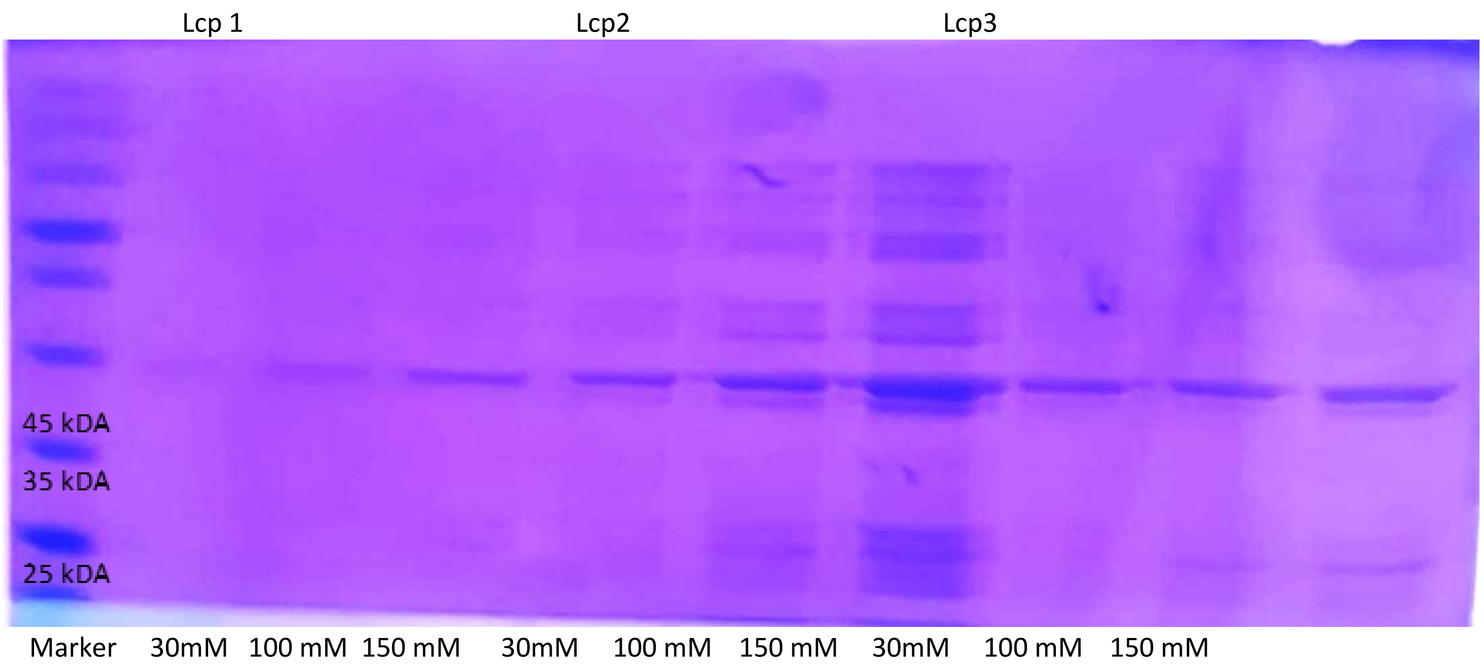

Supplementary Figure 5. SDS-PAGE of purified Lcp (20 mL loaded) for pcold IV using TALON spin column with different concentration of imidazole.
